# Correction of complex three-dimensional deformities at the proximal femur using indirect reduction with angle blade plate and patient-specific instruments: a technical note

**DOI:** 10.1186/s13018-021-02579-z

**Published:** 2021-07-03

**Authors:** Lukas Jud, Lazaros Vlachopoulos, Karl Grob

**Affiliations:** 1 Department of Orthopaedic Surgery, Kantonsspital St. Gallen, Rorschacherstrasse 95, 9007 St. Gallen, Switzerland; 2 Department of Orthopedics, Balgrist University Hospital, University of Zurich, Forchstrasse 340, 8008 Zürich, Switzerland

**Keywords:** 3D printing, Corrective osteotomy, Deformity correction, Hip osteotomy

## Abstract

**Background:**

Corrective osteotomies for complex proximal femoral deformities can be challenging; wherefore, subsidies in preoperative planning and during surgical procedures are considered helpful. Three-dimensional (3D) planning and patient-specific instruments (PSI) are already established in different orthopedic procedures. This study gives an overview on this technique at the proximal femur and proposes a new indirect reduction technique using an angle blade plate.

**Methods:**

Using computed tomography (CT) data, 3D models are generated serving for the preoperative 3D planning. Different guides are used for registration of the planning to the intraoperative situation and to perform the desired osteotomies with the following reduction task. A new valuable tool to perform the correction is the use of a combined osteotomy and implant-positioning guide, with indirect deformity reduction over an angle blade plate.

**Results:**

An overview of the advantages of 3D planning and the use of PSI in complex corrective osteotomies at the proximal femur is provided. Furthermore, a new technique with indirect deformity reduction over an angle blade plate is introduced.

**Conclusion:**

Using 3D planning and PSI for complex corrective osteotomies at the proximal femur can be a useful tool in understanding the individual deformity and performing the aimed deformity reduction. The indirect reduction over the implant is a simple and valuable tool in achieving the desired correction, and concurrently, surgical exposure can be limited to a subvastus approach.

## Background

Proximal femoral osteotomies play an important role in pediatric orthopedic surgery to treat different congenital deformities or childhood disease-related deformities [1–5]. In adults, corrective osteotomies to the proximal femur have become less frequent due to the increasing efficacy of total hip arthroplasty (THA) [6]. However, especially in young patients, such joint-preserving procedures should be considered before performing THA to maintain the native joint, in particular with regard to the high revision rates in such patients [7]. Therefore, different femoral osteotomies still hold their position in the treatment of adults suffering from proximal femoral deformities [8–10]. However, due to the potential complexity of such cases, a detailed preoperative analysis of the individual deformity seems mandatory, and potential technical subsidies during surgery may be useful. Three-dimensional (3D) approaches offer various possibilities in orthopedic surgery, such as the application for training purposes or for preoperative planning of complex and demanding surgical tasks [11, 12]. The preoperative 3D planning can further be enhanced by the combined use with patient-specific instruments (PSI), an approach that has already been established in different orthopedic procedures [13–18]. Zheng et al. [19] previously described the application of 3D-printed navigation templates for locking compression pediatric hip plates (LCP-PHP) in children with femoral neck fractures or hip dysplasia. A recent literature review of Baraza et al. [20] summarized improved accuracy and decreased procedure times in corrective osteotomies of the femur by the application of PSI compared to conventional methods. Overall, an increasing trend of the implementation of 3D planning and the use of PSI at the proximal femur can be observed. Therefore, it was the aim of this study to present an overview of this technique at the proximal femur and to introduce a new technique with indirect deformity reduction using PSI in combination with an angle plate to further improve the toolkit in these demanding surgical tasks.

## Methods

### Preoperative 3D planning

Using computed tomography (CT) data, 3D triangular surface models are generated using already proven segmentation methods [13, 17, 21]. Segmentation in the here-presented cases was performed using commercial segmentation software (Mimics Medical 19.0, Materialise NV, Leuven, Belgium). Regularly, the CT should contain the hip and knee. Especially in cases with large rotational corrections, the ankle can be included to consider the maintenance of the mechanical leg axis in the planning, as such procedures may influence the mechanical leg axis [22]. The 3D models are then imported for the planning into a computer-aided design (CAD) software. For the here-presented cases, the surgical planning software CASPA was used (Balgrist CARD, Zurich, Switzerland). The deformed bone can then be superimposed onto the healthy side as already described for the humerus [15, 23]. The superimposition allows an overview with a better understanding of the underlying deformity and enables an assessment of the needed correction in all planes (Fig. 1). After determining the desired correction, the surgical task can be planned, and the different required PSI can be drafted.

**Fig. 1:**
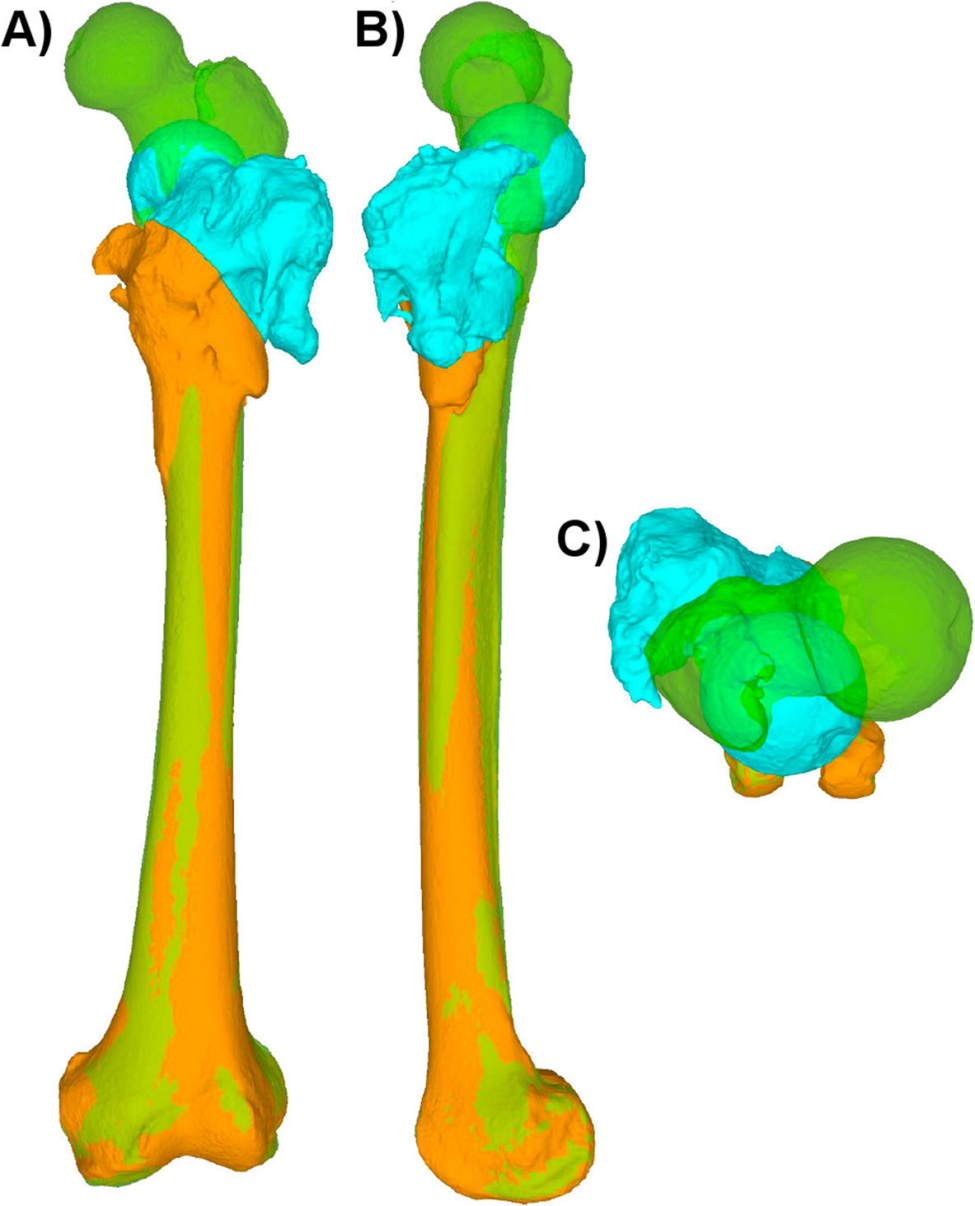
Superimposition of the deformed left femur (orange and turquoise) on the mirrored healthy right side (green). As reference for the superimposition, the distal part of the femur was used (unaffected from the deformity), enabling for a thorough understanding of the deformity of the proximal femur in all three planes (**A** frontal plane, **B** sagittal plane, **C** axial plane)

### Design of patient-specific guides

#### Basic guide

The basic guide is the first used PSI, serving for registration between the preoperative 3D planning and the intraoperative situation. Hence, this guide must contain individual characteristic landmarks in its profile to ensure a precise fit in one specific position. A lateral subvastus approach to the proximal femur is frequently used in corrective osteotomies of the proximal femur [24, 25]. Using such an approach, the trochanteric ridge often serves as a suitable landmark together with the specific circumference of the femoral shaft. The appropriate guide position can further be ensured by integrating stabilizing arms in the guide design [26]. However, removal of the whole soft tissue in the region of the guide storage is still necessary to guarantee the specific guide position. Once the basic guide is located, reference pins can be placed over the integrated drill sleeves. These reference pins serve for setting of all the following guides. To prevent unnecessary bone weakening, the position of these reference pins can be planned in an orientation that these boreholes can be used for later plate fixation (Fig. 2).

**Fig. 2:**
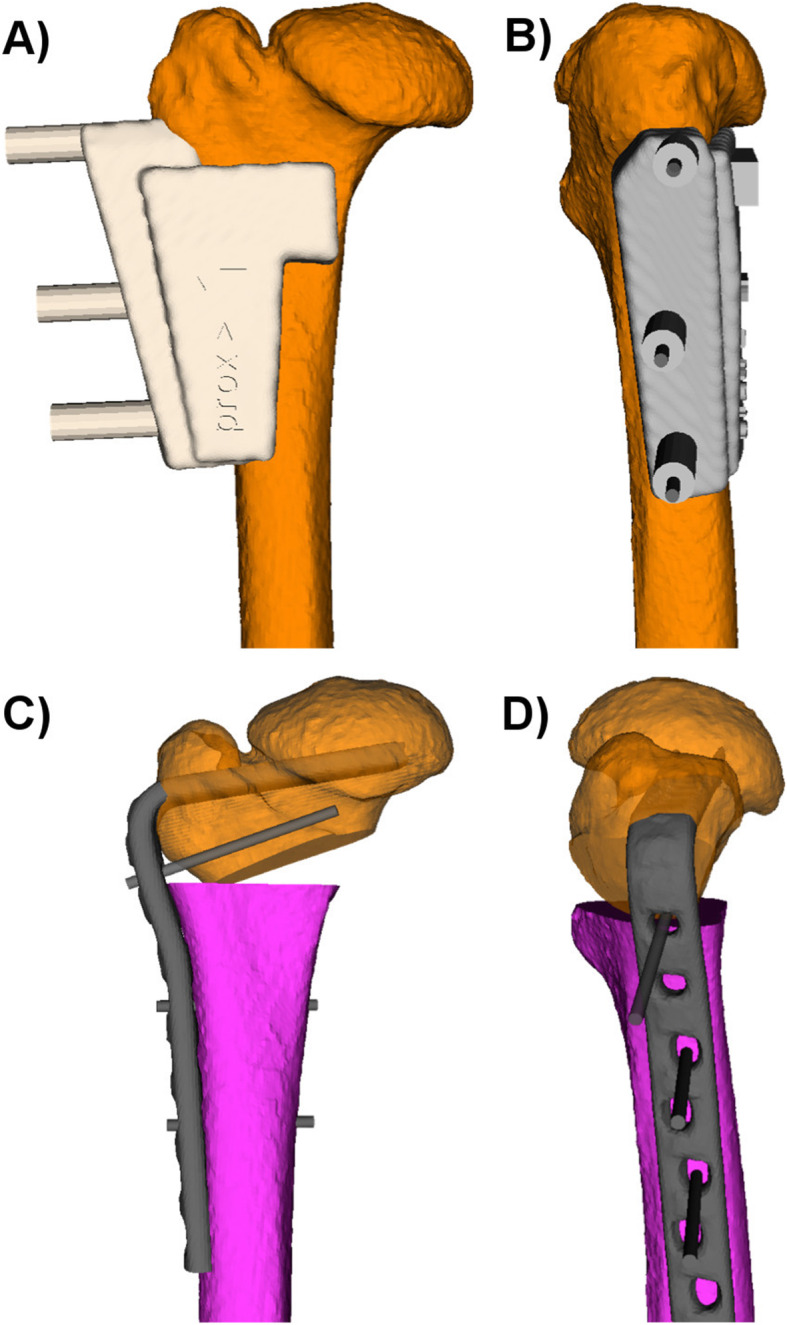
The basic guide (gray) is placed in its specific position (**A** and **B**). In the preoperative planning, the trochanteric ridge and the specific circumference of the femoral shaft were used as characteristic landmarks to integrate them in the undersurface of the guide to find its unique position. The inserted reference pins were planned in an orientation that they correspond to the screw position for later plate fixation (**C** and **D**)

#### Osteotomy and implant-positioning guides

In the next step, the osteotomy guide, respectively, in some cases a combination of an osteotomy and implant-positioning guide can be placed over the reference pins to find its defined position. Most osteotomies at the proximal femur can be performed using an oscillating saw blade. Here, the direction and cutting depth of the saw blade is prescribed by the integrated cutting slit in the guide [15, 16]. However, if a more complex osteotomy is needed, such as a curved osteotomy, this can be achieved using interlinked drill holes, which can be incorporated in the guide design likewise [15]. With regard to the later osteotomy fixation, the placement of the chosen implant can already be considered at this step of the surgery planning. A common implant for fixation of intertrochanteric osteotomies is the use of an angle blade plate [27, 28]. Using such an implant, the blade position in the femoral neck can be defined ahead of the osteotomy using a combination of an osteotomy and implant-positioning guide. Therefore, an additional chisel slit has to be integrated in the guide design (Fig. 3). Same as the cutting slit for the osteotomy, the chisel slit guides the seating chisel in its direction and depth. Additionally, a K-wire hole can be integrated in the guide just above the chisel slit. Placing a K-wire in the defined chisel direction allows C-arm control of the chisel direction ahead of chiseling, serving as a second safety check for an appropriate blade placement. The major advantage of using such a technique is that setting the blade position in the femoral neck before the osteotomy in combination with the predefined screw holes on the femoral shaft (i.e., already placed reference pins using the basic guide) allows later indirect deformity reduction over the angle blade plate. Or in other words, by defining the proximal and distal implant position before the osteotomy, the implant can simply be placed after the osteotomy into the chiseled slit proximally and over the reference pins distally, resulting in the planned deformity correction (Fig. 3). Another advantage of this technique is that the production of an additional reduction guide will become redundant.

**Fig. 3:**
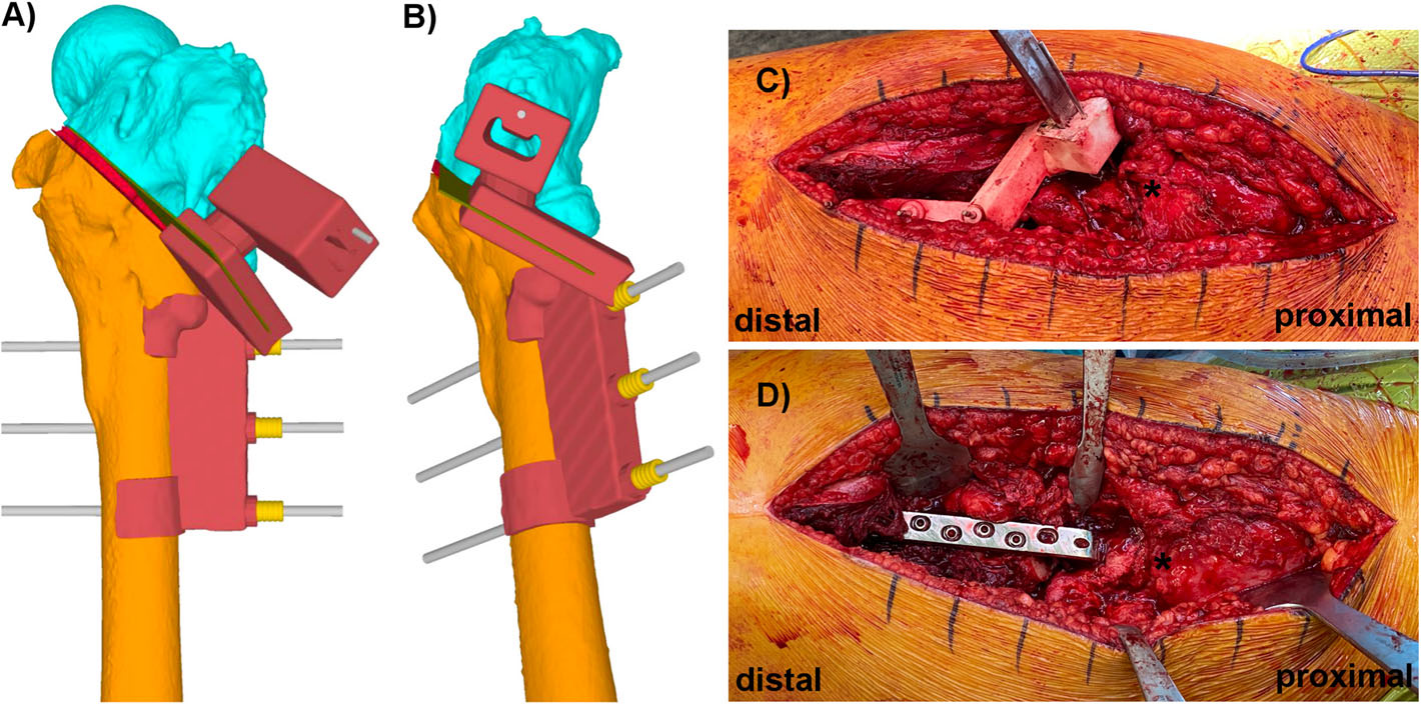
A combination of an osteotomy and implant-positioning guide (**A** and **B** in red) for placement of an angle blade plate. Additional to the cutting slit for the osteotomy (green plane), a chisel slit has been added to the guide design. The chisel slit guides the seating chisel in its direction and depth. A supplementary drilling hole, just above the chiseling slit, has been added for drilling of a K-wire parallel to the chiseling direction. The K-wire serves as a second safety check for the appropriate blade positioning. In **C**, the intraoperative positioned guide with the placed seating chisel, the asterisk marks the greater trochanter. In **D**, the angle plate could simply be placed over the reference pins distally and into the pre-chiseled position proximally after the osteotomy to achieve the aimed deformity correction. The asterisk marks the greater trochanter

#### Reduction guides

Depending on the chosen implant, an indirect reduction over the plate as described above might not be possible. This applies, for example, if the chosen implant is a straight plate like a common locking compression plate (LCP), a frequently used implant for fixation of subtrochanteric rotational osteotomies [22]. In these cases, the deformity correction can be achieved using a reduction guide. This reduction technique is based on direct deformity reduction over the previously placed reference pins. Using this technique, two parallel reference pins are inserted each, proximal and distal from the osteotomy. After the osteotomy, the reduction guide can be placed over the proximal and distal reference pins, aligning them and resulting in the planned reduction of the deformity [15]. Using such a reduction technique, the guide storage of the reduction guide should be planned outside of the desired plate position, to maintain the desired correction by keeping the reduction guide in place during implant positioning (Fig. 4).

**Fig. 4:**
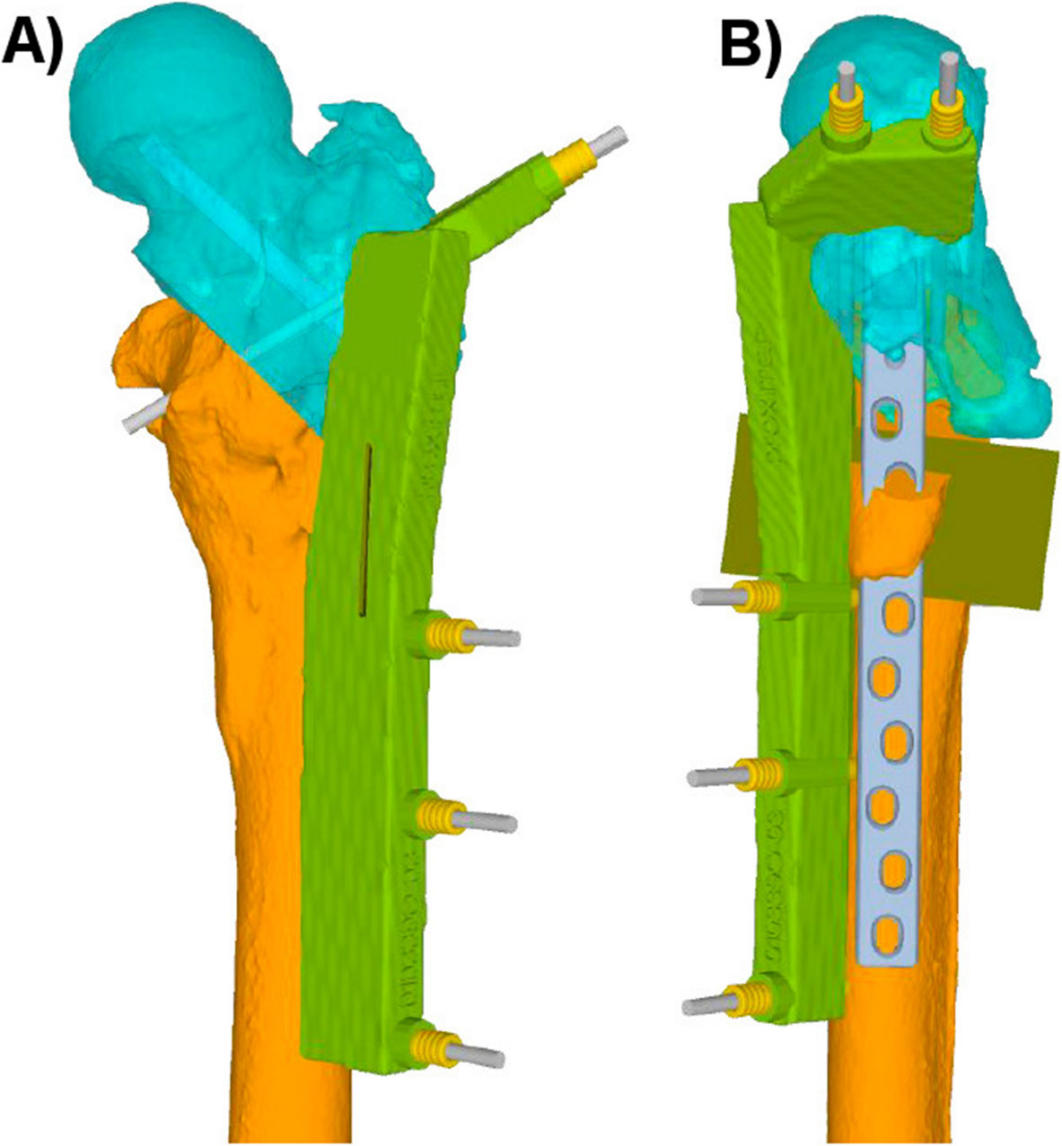
In **A** and **B**, the reduction guide (green) is placed over the reference pins, aligning them and hereby resulting in the aimed deformity correction. An additional osteotomy slit (olive osteotomy plane) is integrated in the reduction guide to prepare the plate storage. The guide storage is planned outside of the intended plate position to maintain the correction by keeping the reduction guide in place during implant fixation

## Discussion

Even though THA shows increasing efficacy, corrective osteotomies in adults retain their position in orthopedic surgery. Beside posttraumatic conditions, non-treated or insufficiently treated congenital conditions or deformities related to childhood diseases may also require corrective osteotomies in adults [8–10, 25]. However, correction of complex deformities at the proximal femur can be challenging [29–31]; wherefore, a detailed preoperative analysis of the deformity and intraoperative subsidies may be useful to achieve the desired deformity correction. The previous literature in the application of 3D planning and the use of PSI in corrective osteotomies at the proximal femur mainly focused on pediatric patients and is lacking the implementation in adults. Furthermore, the indirect deformity reduction over an angle plate using 3D planning and PSI in proximal femoral osteotomies is not yet described. Therefore, the here presented technique is intended to expand the toolkit in planning and correcting deformities at the proximal femur as accurate and valuable as possible. Examples of patients that have undergone corrective osteotomy of the proximal femur using this technique are illustrated in Fig. 5.

**Fig. 5:**
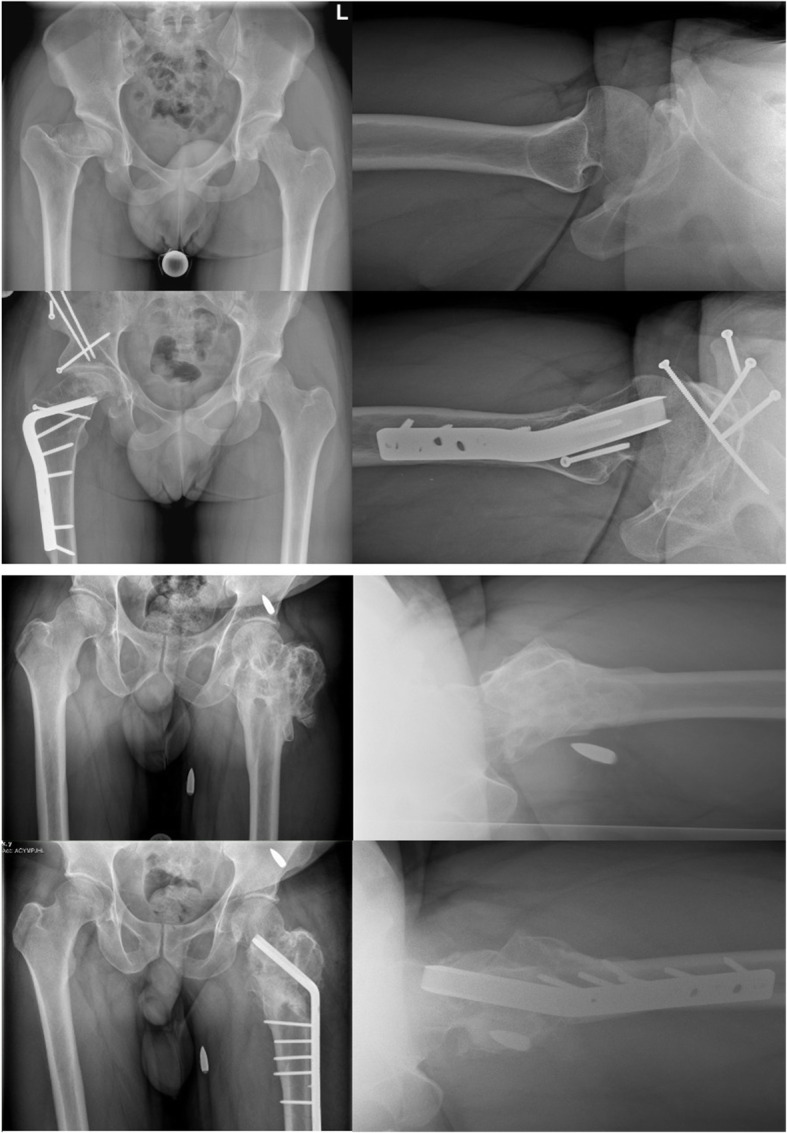
Pre- and postoperative radiographs of patients that have undergone corrective osteotomy at the proximal femur using indirect deformity reduction over the implant and using 3D planning and patient-specific instruments. The patient above had a post-Perthes deformity; he underwent an additional 3D-planned periacetabular osteotomy using patient-specific instruments (surgery performed at Balgrist University Hospital). The patient below had a posttraumatic malunion following a conservatively treated intertrochanteric gunshot fracture of the proximal femur (surgery performed at Cantonal Hospital St. Gallen)

With the ongoing development in orthopedic surgery, the application of 3D technologies is emerging to allow the translation of basic science in clinical practice [32]. The combined use of 3D planning and PSI further enhances its clinical use. The beneficial application of this technique has already been described for corrective osteotomies at different locations [13, 15–17]. At the proximal femur, Zheng et al. [19] showed reduced damage to the femoral neck epiphysis, decreased surgery time, and decreased radiation exposure in the placement of LCP-PHP in children with femoral neck fracture or developmental dysplasia of the hip using 3D planning and PSI. Reduced surgical and fluoroscopy time was also confirmed by Cherkasskiy et al. [33] in triplane proximal femoral osteotomy in children with slipped capital femoral epiphysis using preoperative patient-specific 3D models for surgical planning. Beside reduced radiation exposure and shortened surgery time, Shi et al. [34] also showed improved accuracy using 3D planning and PSI compared to conventional techniques in children with proximal femoral corrective osteotomy in developmental dysplasia of the hip. In a review, Baraza et al. [20] similarly stated improved accuracy in corrective femoral osteotomies using 3D planning and PSI compared to conventional techniques. The improved accuracy probably is a result of the assistance of the PSI, enabling the surgeon in a precise execution of the preoperative planning into the intraoperative situation. Furthermore, the preoperative 3D planning facilitates a better understanding of the underlying deformity, allowing for more accurate preoperative decision-making and probably further improving the surgical accuracy. This objective, in our opinion, seems mandatory as inappropriate preoperative planning or accidental deviations from this planning may result in unintended postoperative results. In addition to the recognized unexpected impact of femoral rotational osteotomies on the mechanical leg axis [35, 36], it is known that mal-angulation of such rotational osteotomies may result in even more considerable mechanical leg axis deviations not only in the frontal plane but also in the sagittal plane [22, 37]. Furthermore, it has to be expected that this undesirable deviations are even more decisive in correcting multiplanar deformities. Therefore, a precise preoperative planning and a surgical execution with the highest accuracy possible seem important especially in cases with complex deformities, requiring corrections in multiple planes.

However, some issues need to be mentioned using the here-described technique. First of all, for the correct guide placement, an appropriate exposure of the characteristic landmarks of the bone is mandatory, and minimal invasive procedures using 3D planning and PSI on the proximal femur are not yet available. However, the invasiveness of the surgery can still be kept low due to the detailed preoperative planning with a minimum number of boreholes (due to the fact that the boreholes for the guides already serve for later implant placement) and the integrated cutting/chiseling slits on the PSI direct the surgeon in the desired orientation without further extensive exposure, as it is needed to control for orientation of the osteotomy or the implant placement in some conventional procedures. Furthermore, with the here-described indirect reduction technique, further extensive soft tissue removal for placement of additional guides outside of the implant storage (e.g., reduction guide) gets redundant, and the surgical exposure can be limited to a subvastus approach. Therefore, surrounding tissue can be preserved compared to previous techniques. Another issue is the financial aspect and the expenditure of time for planning. An appropriate preoperative 3D planning and the production of the PSI result in additional costs and can be time-consuming. Therefore, for each individual case, it has to be weighed out if the benefits from this technique justify these costs and the additional time requirement. Probably especially in young patients with complex deformities requiring multiplanar corrections, these additional efforts should be warranted. An overview on the advantages and disadvantages of the here-described technique is provided in Table 1.

**Table 1:** Overview on the advantages and disadvantages using 3D planning and patient-specific instruments with the here-described technique

Advantages	Disadvantages
↑ Deformity understanding	↑ Costs
Individual planning	↑ Time for planning
Limited exposure due to guidance	Minimally invasive techniques not yet available
Simple deformity correction using indirect reduction technique	

## Conclusion

Using 3D planning and PSI for complex corrective osteotomies at the proximal femur can be a useful tool in understanding the individual deformity and performing the aimed deformity reduction. The indirect reduction over the implant is a simple and valuable tool in achieving the desired correction, and concurrently, surgical exposure can be limited to a subvastus approach.

## Data Availability

Anonymized source data can be obtained from the corresponding author on reasonable request.

## Abbreviations

3D: Three-dimensional
PSI: Patient-specific instruments
CT: Computed tomography
THA: Total hip arthroplasty
LCP-PHP: Locking compression pediatric hip plates
CAD: Computer-aided design
LCP: Locking compression plate
